# PRB1 Is Required for Clipping of the Histone H3 N Terminal Tail in *Saccharomyces cerevisiae*

**DOI:** 10.1371/journal.pone.0090496

**Published:** 2014-02-28

**Authors:** Yong Xue, Ajay A. Vashisht, Yuliang Tan, Trent Su, James A. Wohlschlegel

**Affiliations:** Department of Biological Chemistry, David Geffen School of Medicine, University of California Los Angeles, Los Angeles, California, United States of America; Simon Fraser University, Canada

## Abstract

Cathepsin L, a lysosomal protein in mouse embryonic stem cells has been shown to clip the histone H3 N- terminus, an activity associated with gene activity during mouse cell development. Glutamate dehydrogenase (GDH) was also identified as histone H3 specific protease in chicken liver, which has been connected to gene expression during aging. In baker’s yeast, *Saccharomyces cerevisiae*, clipping the histone H3 N-terminus has been associated with gene activation in stationary phase but the protease responsible for the yeast histone H3 endopeptidase activity had not been identified. In searching for a yeast histone H3 endopeptidase, we found that yeast vacuolar protein Prb1 is present in the cellular fraction enriched for the H3 N-terminus endopeptidase activity and this endopeptidase activity is lost in the *PRB1* deletion mutant (*prb1*Δ). In addition, like Cathepsin L and GDH, purified Prb1 from yeast cleaves H3 between Lys23 and Ala24 in the N-terminus *in vitro* as shown by Edman degradation. In conclusion, our data argue that PRB1 is required for clipping of the histone H3 N-terminal tail in *Saccharomyces cerevisiae*.

## Introduction

Eukaryotic chromosomal DNA is packaged in nucleosomes which contain 2 copies each of the core histones H2A, H2B, H3 and H4 [1]. The core histones are highly conserved in evolution from yeast to humans and are heavily modified not only at the N termini that extend from their globular cores but also at the globular domains themselves. This provides functional variation to these small highly basic proteins. There are also specialized variant histones that are distinguishable from the canonical histones by differences in primary structure. Histone modifications and variant histones play important roles in numerous cellular processes including DNA replication, gene expression and aging [2], [3], [4], [5].

The histone H3 N-terminal tail is the target of many such modifications that are involved in gene activity [6], [7], [8], silencing of transcription and higher order structure of heterochromatin [9], [10], [11], replication [12], nucleosome assembly [13], [14] and chromatin remodeling [15]. Interestingly, a major H3 modification is the conserved proteolytic cleavage in the histone H3 N-terminus. For example, in *Saccharomyces cerevisiae*, a serine protease activity is present that cleaves the histone H3 tail after Ala21 in sporulation and stationary phase, and preventing truncation using a mutation H3 Q19A, L20A results in decreased transcription of selected yeast genes in stationary phase [16]. In chicken, a tissue-specific histone H3 N-terminal cleavage activity was found in adult chicken liver that has been connected to gene expression during aging [17] and later it was identified as GDH (Glutamate dehydrogenase) which cleaves H3 between Lys23 and Ala24 [18], [19]. Finally, mouse cathepsin L, a lysosomal cysteine protease, is required for the cleavage of H3 tails between residues 21–27 during embryonic stem cell differentiation [20]. The presence of the H3 tail-truncating serine protease activity in *S. cerevisiae* has stimulated the search for the enzyme responsible for clipping the H3 tail. However, a search of 21 viable deletion strains lacking different serine proteases found that none of the deletion strains lost the ability to clip H3, prompting the suggestion that redundant enzymes were involved [16].

In the report below, we now describe the purification of a histone H3 N-terminus endopeptidase activity from *S. cerevisiae* through a traditional biochemical approach. We found that the vacuolar proteinase B (Prb1) is present in the active fraction purified from *S*. *cerevisiae* whole cell extracts. Additionally, the H3 endopeptidase activity was lost in PRB1 deletion mutant. Our data revealed that *PRB1* is required for clipping the histone H3 N-terminus in both whole cell extracts and nuclear extracts. Furthermore, we found that purified Prb1 cleaves the H3 N terminus between Lys23 and Ala24 *in vitro*. Taken together, our data argue that the vacuolar serine proteinase Prb1 is required for the H3 N-terminus truncation in yeast.

## Materials and Methods

### Yeast Strains, Plasmids and Media

Yeast strains and plasmids used in this work are described in Table S1. Standard yeast media and manipulations were used. The protocol for gene knockout was described earlier [21]. Plasmids carrying histone mutations were first generated from plasmid pNS001 using the QuickChange site-directed mutagenesis kit (Stratagene). Histone mutations were confirmed by sequencing. These plasmids were further digested with restriction enzyme (BamHI and SalI from NEB) and the mutated histone fragments were ligated into plasmid pRM200 [6] to obtain the final histone mutation plasmids. Plasmid for expression of recombinant *Xenopus laevis* histone H3 was a gift from Michael Carey. Plasmid pBH105 for the expression of recombinant *S. cerevisiae* histone H3 was provided by Bing Li.

### Yeast Histone H3 Endopeptidase Purification

Yeast colonies (RMYT200) were inoculated into 200 ml YPD medium and incubated at 30°C overnight. The overnight culture was diluted to OD_600_ of 0.2 in 2 L YPD medium. After 24 hours incubation at 30°C, the early stationary phase cells were collected by centrifugation. Spheroplasts were prepared as described [22] except that we used yeast lytic enzyme (Fisher, BP2683-25) at 37°C for 1 hour. Spheroplasts were lysed in lysis buffer (20 mM Tris-HCl, pH 8.5, 200 mM KCl, 25 mM EDTA, 1% (v/v) Triton X-100) on ice for 20 min. The supernatants were collected by centrifugation and used as whole cell extracts. The purification of histone H3 endopeptidase was followed by chromatography on anion exchange (Pharmacia, 17-0556-01, Mono Q HR10/10, with or without NaCl in buffer 20 mM Tris-HCl pH 7.4, 1 mM EDTA, 10% Glycerol, 0.01% NP-40), gel filtration (Pharmacia, 17-1047-01, SuperdexTM 75 HR10/30, 20 mM Tris-HCl pH 7.4, 1 mM EDTA, 150 mM NaCl) and hydrophobic interaction (GE, 17-1351-01, HiTrapTM Phenyl SP, 20 mM Tris-HCl pH 7.4, 1 mM EDTA with ammonium sulfate gradient).

### Endopeptidase Reactions

Recombinant *X. laevis* and *S. cerevisiae* histone H3 were prepared as described previously [23]. Prc1(Carboxypeptidase Y) was purchased from Sigma Aldrich(C3888-1MG). To purify the H3 endopeptidase, the column fractions were incubated with 1–2 µg recombinant *X*. *laevis* histone H3 in reaction buffer (20 mM Tris-HCl, pH 7.4, 1 mM EDTA, 150 mM NaCl) at 30°C for 1 hour with shaking. Proteins were separated by SDS-PAGE, stained with Ponceau S and analyzed by western blot using anti H3-C terminus antibody (ab1791). To determine whether Prb1 is required for the H3 endopeptidase activity in vivo, the endopeptidase reactions were performed by incubating either whole cell extracts or nuclei with 1 µg recombinant *X. laevis* histone H3 at 30°C for 2 hours with shaking. To determine the cleavage site of histone H3 by nuclei extracts or purified Prb1, the endopeptidase reactions were performed by incubating either nuclei or purified Prb1 with 1–2 µg recombinant *S. cerevisiae* histone H3 at 30°C for 1 hour with shaking. 2 mM PMSF and 1.5 µM Pepstatin A as proteinase inhibitor were used when indicated.

### Antibodies

Anti H3 C-terminus antibody (ab1791) was purchased from Abcam. Anti HA antibody (12CA5) was purchased from Roche. Anti H3 truncation antibody (3008-72) was generated at Pi Proteomics Inc using a branched peptide NH2-(SKAAR)_2_-KC-Amide. The antibody was validated by ELISA with control peptide NH2-RKQLASKAARKC-Amide by the same company. For western blot, the antibody was further purified using the Sulfolink Immobilization Kit (Thermo Scientific/44999) following the manufacturer’s protocol.

### Western Blot

Histones were separated using SDS-PAGE and transferred to a nitrocellulose membrane using the iBlot Dry Blotting System (Life Technologies) following the manufacturer’s protocol. The membranes were blocked with TBS blocking buffer (Thermo scientific/37543) for 1 hour at room temperature, then incubated in primary antibody at 4°C overnight and secondary antibody for 1 h at room temperature. The following dilutions were used for primary antibody incubation: 1∶50000 H3 (ab1791), 1∶1000 HA (12CA5), 1∶500 tH3 (3008-72).

### Mass Spectrometry and Matrix-assisted Laser Desorption/Ionization Analysis

The fractions or purified proteins were precipitated using TCA. TCA precipitates were resuspended in digestion buffer (100 mM Tris-HCl, pH 8.5, 8 M urea), digested by the sequential addition of Lys-C and trypsin proteases, fractionated online using a C18 reversed phase column, and analyzed by MS/MS on a Thermofisher LTQ- Orbitrap XL as previously described [24], [25]. MS/MS spectra were subsequently analyzed using the ProLuCID and DTASelect algorithms [26], [27].

### Preparation of Yeast Whole Cell Extract (WCE) and Nuclei

Early stationary phase yeast cells were prepared as described before. A total of 40 OD_600_ cells were resuspended in 400 µl lysis buffer and vortexed with 50% glass beads for 45 minutes at 4°C. The lysates were collected in a 1.5 ml eppendorf centrifuge tube by puncturing the bottom of the tube with a flamed needle followed by spinning into a second tube in an eppendorf microcentrifuge. The supernatant obtained after further spin were used as whole cell extracts (WCEs). The WCEs were diluted to 40 fold with reaction buffer and 1 µl was used for the endopeptidase reactions in a 10 µl reaction. Nuclei for the H3 endopeptidase activity were prepared as described previously [28] without any protease inhibitor. The nuclear fraction for the measurement of endogenous truncated H3 level was prepared as described previously [22].

### Purification of Prb1 from Yeast

Cells from the Yeast ORF collection library (Open Biosystems) were first incubated in synthetic raffinose medium (SR) (for WT, Y258) or SR-URA (for PRB1, YSC3869-9518684) to OD_600_ 0.6–0.8 and induced with 2% galactose for 6 hours. The cells were collected by centrifugation, resuspended in an equal volume of TAP buffer (40 mM HEPES, pH 7.5, 10% Glycerol, 350 mM NaCl, 0.1% Tween-20) and vortexed with 50% glass beads for 45 minutes at 4°C. The lysates were collected by puncturing the bottom of a tube as described above followed by high speed centrifugation after treatment with DNase I and Heparin each for 5 min at room temperature. The protein was first purified by using IgG sepharose beads and TEV protease as previously described with minor modifications [29] and further purified with Ni-NTA Agarose (QIAGEN) using binding buffer (20 mM NaH_2_PO4, pH 7.4, 0.5 M NaCl, 10 mM Imidazole) and elution buffer containing 500 mM Imidazole. Finally, protein was dialyzed and concentrated in reaction buffer including 10% glycerol. 1 mM benzamidine was added during the whole purification except for the last step-dialysis and concentration.

## Results

### Prb1 is Present in the Chromatographic Fractions Enriched with Histone H3 N-terminus Endopeptidase Activity

**Figure 1:**
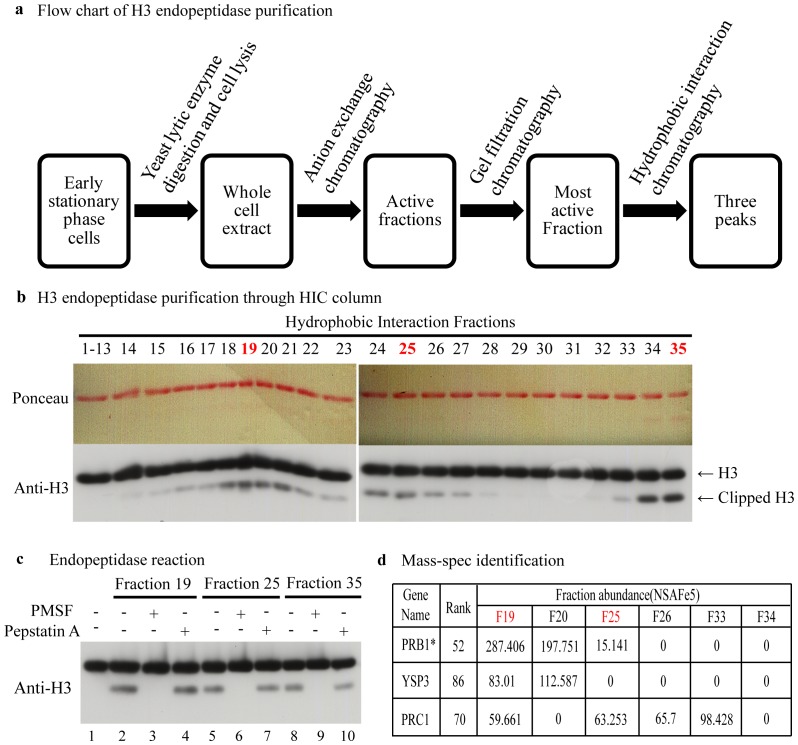
Identifying the histone H3 endopeptidase. (a) Schematic of histone H3 endopeptidase enrichment. Whole cell extract from early stationary phase cells was used to purify the histone H3 endopeptidase. Endopeptidases were purified by chromatography by Anion exchange, Gel filtration and Hydrophobic Interaction. The cleavage activity was measured by western blot with antibody against the H3 C-terminus. (b) H3 endopeptidase assay of the fractions separated by Hydrophobic Interaction Chromatography. The red numbers represent the peaks of cleavage activity (analyzed by Image J). (c) Yeast histone H3 endopeptidases are serine proteases: The H3 endopeptidase activity of Fractions 19, 25 and 35 was assayed with and without protease inhibitors and clipping was assayed by western blot with antibody against the H3 C-terminus. (d) Mass spectrometry analysis of serine protease candidates (Prb1, Ysp3, Prc1) in fractions that include the peaks of endopeptidase activity.

To identify and characterize yeast histone H3 N-terminus endopeptidases, we used traditional biochemical methods to purify the protease from whole cell extracts of early stationary phase cells. The endopeptidase activity was detected by following the previous protocol [16] except using recombinant *Xenopus laevis* histone H3 instead of recombinant human H3.1 or calf thymus H3. Antibody raised against the H3 C-terminus was used to identify H3 truncated by the endopeptidase at its N terminus. Histone H3 endopeptidases were first enriched by anion exchange chromatography. Active fractions (elution between 0–0.36 M NaCl) were collected and further purified by gel filtration chromatography and fractions containing peak activity were finally separated by hydrophobic interaction chromatography (HIC). The flow chart of the purification process is shown in Figure 1a. We found that three peaks of cleavage activity were present in the fractions separated by HIC as shown in Figure 1b (peak fractions are labeled in red) and that all of them can be inhibited by the serine protease inhibitor PMSF (Figure 1c, lanes 3, 6 and 9) but not by the aspartyl protease inhibitor Pepstatin A (Figure 1c, lane 4, 7 and 10). Therefore, our findings agree with previous studies by demonstrating that the yeast H3 N-terminus endopeptidase activity comes from a serine protease and its endopeptidase activity can be inhibited by PMSF [16].

To further characterize the yeast H3 endopeptidases, we used Mass Spectrometry (MS) to identify proteins in the fractions around peaks of cleavage activity (fractions 19, 20, 25, 26, 33 and 34). The abundance of proteins was expressed in units of NSAF (normalized spectral abundance factor) [30]. In total, 174 proteins were identified and of which three (Prb1, Prc1 and Ysp3) are serine proteases (Table S2). Prb1 and Prc1 are both vacuolar proteinases and the function of Ysp3 is unknown. We also compared the abundance of these three serine proteases within different fractions with H3 cleavage activity. As shown in Figure 1d, more Prb1 is detected by MS in fraction 19 (287) than in fraction 20 (197). We found that the abundance of Prb1 correlates well with the level of cleavage activity in fractions 19 and 20, while the abundance of Prc1 and Ysp3 correlates less well (Figure 1b and Figure 1d). These data indicate that Prb1 and to a lesser extent, Prc1 and Ysp3, are candidate proteins with the H3 endopeptidase activity.

### PRB1 is Required for Histone H3 N-terminus Endopeptidase Activity *in vivo*

**Figure 2:**
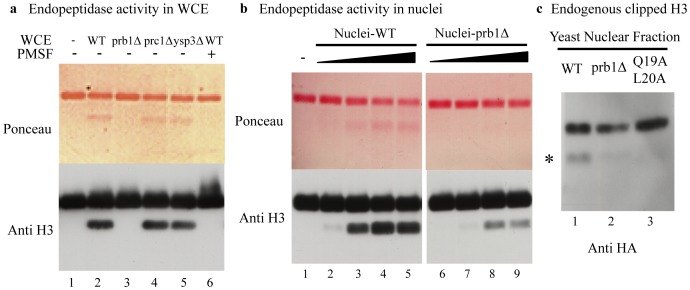
PRB1 is required to cleave histone H3 at its N-terminus *in vivo.* (a) *PRB1* is essential for the H3 endopeptidase activity in whole cell extracts of early stationary phase cells. Whole cell extracts from WT, *prb1*Δ, *prc1*Δ and *ysp3*Δ were assayed for endopeptidase activity. Clipping was assayed by western blot with antibody against the H3 C-terminus. (b) *PRB1* is required for H3 endopeptidase activity in nuclei. Nuclei from WT and *prb1*Δ cells were assayed for endopeptidase activity. Clipping was assayed by western blot with antibody against the H3 C-terminus. The nuclei gradient is represented by 4 fold dilutions. (c) Endogenous truncated histone H3 level was decreased in the *prb1*Δ strain. Nuclear fractions were purified from WT, *prb1*Δ and H3 Q19A, L20A mutant strains in early stationary phase and truncated H3 levels were measured by western blot with antibody against the HA tag.

To test whether one or more of the three serine proteases (PRB1, PRC1 and YSP3) is required for H3 endopeptidase activity in vivo, we deleted each gene in our yeast strain (YXY035) as described in the Material and Methods. Whole cell extracts (WCEs) of deletion mutants in early stationary phase was used to measure the H3 endopeptidase activity. As shown in Figure 2a, using the antibody against the H3 C terminus, we found that an activity WCEs of WT cells can cleave added *X. laevis* H3 at its N-terminus (Fig. 2a, lane 2) and that the activity can be inhibited by PMSF (Fig. 2a, lane 6). In addition, the cleavage activity is lost in the *prb1*Δ mutant (Fig. 2a, lane 3 compared to lane 2) while *prc1*Δ and *ysp3*Δ mutants showed no apparent decrease in cleavage activity when compared to WT (Fig. 2a, lane 4 and 5 compared to lane 2). These data indicate that *PRB1* is required for H3 endopeptidase activity in vivo.

Since it has been shown that histone H3 endopeptidase activity is present in yeast nuclei [16], we also searched for Prb1 endopeptidase activity in yeast nuclei isolated as previous described [16]. We confirmed that an endopeptidase activity in the yeast nuclear extract from WT cells cleaved recombinant *X. laevis* histone H3 (rH3) at its N-terminus (Figure 2b, lane 2–5). In contrast, the endopeptidase activity in the nuclear extract isolated from *prb1*Δ cells was strongly reduced (Figure 2b, lanes 6–9 compared to lanes 2–5) indicating that *PRB1* is required for the H3 endopeptidase activity in nuclei. Additionally, our data suggests that there are other H3 endopeptidases present in the nuclei since the nuclear H3 endopeptidase activity was not completely abolished by *prb1*Δ (Fig. 2b lanes 8 and 9). This funding is also consistent with previous results that there are three peaks of H3 endopeptidase activity separated by HIC (Fig. 1b).

To ask whether the truncation of endogenous yeast histone H3 is also dependent on *PRB1*, we examined the truncated H3 level in the purified nuclear fraction from WT, *prb1*Δ and H3 Q19A L20A mutant strains. To detect endogenous yeast histone H3, we fused an HA tag to the C terminus of H3 and introduced this construct into the strains used for obtaining the nuclear extracts. As shown in Figure 2c, by using anti-HA antibody, we found that H3 was truncated at its N terminus in our WT strain (Fig. 2c, lane 1) and that the level of truncated H3 was markedly reduced in the *prb1*Δ mutant (Figure 2c, lane 2 compared to lane 1). As expected, the level of truncated H3 decreased in the H3 Q19A, L20A mutant (Figure 2c, lane 3) which has been shown to reduce truncation of H3 in yeast (16). Therefore, *PRB1* is required for the nuclear endopeptidase activity that clips yeast histone H3 in vivo.

### Purified PRB1 from Yeast Cleaves the Histone H3 N-terminus *in vitro*

We then wished to determine whether Prb1 purified from yeast cells has H3 endopeptidase activity. The *PRB1* gene in *S. cerevisiae* encodes the 69.6 kDa vacuolar proteinase B precursor. During its secretion, the precursor gets processed post-translationally at both its N terminus and C terminus into a 31 kDa active PrB protease [31] containing amino acids 281–573 from the full-length protein. Also included in this mature protein is a 2.9 kDa domain of non-Asn-linked carbohydrate [31], [32]. Due to the difficulty of Prb1 refolding correctly from denatured form, we switched to purifying Prb1 in yeast after many failed attempts to purify the active mature form of recombinant Prb1 from E.coli. To purify mature Prb1 in yeast, we used a different approach that utilized the strain YSC3869-9518684 obtained from the Yeast ORF collection library (Open Biosystems). This strain contains the *PRB1* expression plasmid from which *PRB1* is expressed under P_GAL_ promoter control starting at the natural N-terminal methionine and ending with a fusion of the C-terminal amino acid to a tag consisting of His6, an HA epitope, a protease 3C cleavage site, and the IgG-binding domain from protein A [33]. *PRB1* expression was induced in the presence of galactose in log phase cells and Prb1 was first purified using IgG Sepharose beads and TEV protease treatment. Prb1 was then purified further on Ni-NTA Agarose beads. The host strain (without plasmid) was processed at the same time and used as a control.

**Figure 3:**
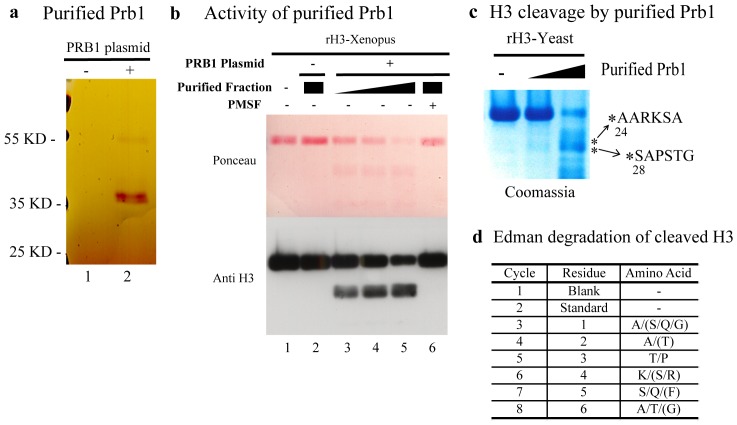
Purified Prb1 can cleave recombinant histone H3 at its N-terminus *in vitro.* (a) Purification of Prb1 from yeast demonstrated by silver staining. Endopeptidase was purified from yeast with or without the PRB1 expression plasmid as described in the Materials and Methods. The protease was separated by SDS-PAGE and stained with silver. (b) Prb1 cleaves recombinant *X. laevis* histone H3 at its N-terminus in vitro. Purified Prb1 was assayed for endopeptidase activity with recombinant *X. laevis* histone H3. Purified protease from host strain (without plasmid) was used as control. (c) and (d) Prb1cleaves recombinant *S. cerevisiae* histone H3 between Lys23/Ala24 and Lys27/Ser28. (c) Recombinant *S. cerevisiae* histone H3 was incubated with purified Prb1, then H3 was separated by SDS-PAGE, transferred to PVDF and stained with Coomassie blue. The bands labeled with asterisk were excised and subjected to Edman degradation. (d) Results of Edman degradation sequencing. Cycle 1 is a blank run. Cycle 2 is the amino acid standard. Cycles 3–8 represent the six cycles of the clipped H3 corresponding to the N-terminal amino acid. X/Y/Z means that 2 or more residues were identified in each cycle. (X/Y) means that the identification of some residues was uncertain.

Silver staining of the purified fraction showed one major band of ∼37 kDa (Figure 3a, lane 2), which was processed to ∼ 31 kDa after 1 hour incubation at room temperature (Figure S1a). The purified fraction from the host strain did not show the presence of any band (Figure 3a, lane 1). Our data is consistent with the processing of Prb1 from the 37 kDa intermediate to the mature 31 kDa Prb1 protein [31]. MS analysis also confirmed that Prb1 is the most abundant protein in the purified fraction which contained no other known proteases (Figure S1b). We then asked whether purified Prb1 could cleave H3 at its N-terminus *in vitro*. After incubating recombinant *X. laevis* histone H3 with purified Prb1, we found that H3 was cleaved at its N-terminus using the antibody against H3 C terminus (Figure 3b, lanes 3, 4 and 5) and that the cleavage activity of Prb1 was inhibited by PMSF (Figure 3b, lane 6). As control, the fraction purified from the host strain (without plasmid) did not show any cleavage activity (Fig. 3b, lane 2), which indicates that the cleavage activity is dependent on Prb1 purification. Taken together, these data argue that purified Prb1 can clip recombinant *X. laevis* H3 at its N terminus in vitro.

To further determine the site in yeast H3 clipped by Prb1, we incubated purified Prb1 with recombinant yeast H3. Cleaved H3 was separated by SDS-PAGE, transferred to PVDF membrane and subjected to Edman degradation, which was performed by Alphalyse Inc. As shown in Figure 3c and 3d, the result of Edman degradation strongly suggests that the primary purified Prb1 cleavage site in yeast H3 in vitro is between Lys23 and Ala24. An additional but less abundant Prb1 cleavage site in H3 is located between Lys27 and Ser28. Interestingly, GDH and Cathepsin L also cleavage histone H3 at the same sites (Lys23/Ala24 and Lys27/Ser28) [18], [19], [20] which is consistent with our result. We also detected some intact H3 N-terminal sequence (ARTKQT) in truncated H3, which indicates that a fraction of truncated H3 resulted from cleavage at the C terminus but we did not see cleavage in vitro of recombinant yeast H3 between Ala21 and Ser22.

In contrast, a previous study showed that truncated H3 isolated from purified yeast chromatin in early stationary phase starts at Ser22 [16]. To investigate whether some regulators are involved which could affect the Prb1 cleavage site on H3, we decided to compare the H3 cleavage activity obtained with purified Prb1 to the activity found in the nuclear fraction. To accomplish this, we first generated an antibody (3008-72) which was raised against the clipped H3 starting at Ser22 as described in the Materials and Methods. To validate the antibody we constructed a H3 1-21Δ yeast mutant strain which mimics clipped histone H3 starting at Ser22. The WCEs of log phase cells from WT and H3 1-21Δ mutant strains were separated by SDS-PAGE and H3 cleavage levels was assayed by western blot. As shown in Figure S1c, the 3008-72 antibody recognizes the clipped H3 starting at Ser22 (Figure S1c lane 2, H3 1-21Δ) but not full length H3 (lane 1). As controls, both full length H3 and H3 1-21Δ carrying an HA tag at their C termini were detected using anti HA antibody (lanes 3 and 4). After confirm specificity of the antibody, we then assayed for H3 endopeptidase cleavage of the added recombinant yeast H3 using yeast nuclear extracts or purified Prb1. As shown in Figure S1d, when using the anti H3-C terminus antibody (middle panel), both the nuclear fraction and purified Prb1 can cleave H3 at its N-terminus. However, when using anti H3 truncation antibody 3008-72 for detection, it is evident that cleavage between residues 21 and 22 (Figure S1d III, starred band) occurs only when H3 is treated with the nuclear extract (Figure S1d III, lane 2 and 3). These findings confirm the presence of H3 endopeptidase activity cleaving H3 after residue 21 in the nucleus. Furthermore, some other cellular regulators besides PRB1 may be involved in determining the specificity of the cleavage site on H3.

## Discussion

We have identified yeast vacuolar protein Prb1 to be required for endogenous nuclear H3 endopeptidase activity. Additionally, it is responsible for majority of the cleavage events occurring at histone H3 N terminus in stationary phase. The activity of Prb1 is known to be repressed by glucose and derepressed when cells are in stationary phase or undergoing sporulation [34], [35], [36], [37]. This is consistent with the presence of the previously identified yeast H3 endopeptidase activity [16]. Even though WCES of *prb1*Δ cells have dramatic reduction in H3 clipping activities, the HIC fractions containing Prb1 (19/20) did not demonstrate the strongest H3 endopeptidase activity on recombinant *Xenopus laevis* histone H3 in vitro when compared to HIC fractions 34/35. We speculate that a lot of Prb1 in HIC fraction 19/20 may have already been degraded after chromatography on anion exchange and gel filtration. Previous studies did showed that purified mature Prb1 is very unstable [38], [39], [40]. Additionally, the presence of H3 endopeptidase activity in nuclei of prb1Δ indicated that Prb1 is unlikely to represent the full repertoire of H3 endopeptidases. Therefore, it is likely that other redundant H3 endopeptidase exist in yeast. Aside from Prb1, there are two other serine proteases belonging to the same subfamily in *S. cerevisiae*: *RRT12* and *YSP3*. However we did not find *RRT12* in MS analysis of our purified endopeptidase fractions and deletion of *YSP3* did not affect histone H3 endopeptidase activity in whole cell extracts. Glutamate dehydrogenase (GDH) was identified recently as histone H3 specific serine protease in chicken liver. There are three genes (GDH1, GDH2 and GDH3) encoding glutamate dehydrogenase in budding yeast. Interestingly, GDH1 was identified fractions 19/20. However, the single enzyme including GDH1 knockout mutant strains from the yeast knockout library (Thermo Scientific) doesn’t decrease the H3 endopeptidase activity in WCEs (Figure S2a). Perhaps the GDH enzymes have redundant activities not observable in single deletions of any one enzyme. We also noticed that Prb1, Ysp3 and GDHs is absent in HIC fractions 33/34 and purified Prc1 (Sigma Aldrich) did not cleave H3 at its N terminus (Figure S2b). These data indicate that at least aother novel H3 endopeptidase is present in the last peak from HIC which is worth to pursue in the future.

The identification of *PRB1* as an H3 N-terminus clipping enzyme extends the function of Prb1 which is generally involved in protein degradation in the vacuole. However, a number of findings support the nuclear localization of H3 clipping enzymes. First, nuclear localization also appears to be a feature of cathepsin L, a lysozomal endopeptidase [41], [42], that cleaves histone H3 during stem cell differentiation [20]. Second, sequence analysis predicts that Prb1 has a nuclear localization signal in the mature form. (Predicted from http://wolfpsort.org/). Third, vacuoles can bind nuclei via nucleus-vacuole (NV) junctions in stationary phase cells [43] which provides the opportunity for Prb1 to enter nuclei. Finally, the H3 endopeptidase activity and truncated H3 level from purified nuclei was decreased in the *PRB1* deletion strain in our study (Fig. 2, b and c). Nevertheless, we cannot exclude the possibility that some of the nuclear H3 clipping activity we have purified may have come from vacuolar contamination during purification of nuclei. Direct evidence for the localization of Prb1 to nuclei would require chromatin immunoprecipitation (ChIP) or Immunofluorescence with anti-Prb1 antibody.

We have shown that purified Prb1 cleaves yeast histone H3 between Lys23 and Ala24 but the *PRB1* dependent nuclear H3 endopeptidase activity cleaves H3 between Ala21 and Ser22. So it is unlikely that H3 is cleaved between Lys23 and Ala24 in vivo though Prb1 does in vitro. While these sites of cleavage are close to each other it is interesting to speculate why H3 in yeast chromatin is cleaved N terminal at certain clipping site. Amongst other possibilities, this may be a result of the protection of on both Lys23/Ala24 at N terminal and C terminal in yeast chromatin, and the involvement of regulators which target Prb1 to Ala21/Ser22 in chromatin. It is also formally possible that Prb1 is required for the activity of yet another unidentified nuclear serine protease which cleaves H3 at Ala21/Ser22. The detailed mechanism that how the H3 is been cleaved at specific site need to be determined in the future.

In conclusion, *PRB1* was shown here to be required for H3 N terminal clipping in yeast. Its identification should be useful in determining the full spectrum of H3 N-terminal endopeptidases and their roles in gene regulation.

## Supporting Information

Figure S1 **Validation of the purified Prb1, antibody t3008-72 and comparison of the cleavage site of H3 by purified Prb1 and nuclei extract.** (a) Proteolysis of purified Prb1. Purified Prb1 was incubated either on ice or room temperature for 1 hour, then separated by SDS-PAGE and stained by silver. (b) Mass spectrometry analysis of the purified Prb1. Prb1 was purified from the strain YSC3869-9518684 (Open Biosystems) containing the PRB1 expression plasmid as described in the Materials and Methods. The abundance of proteins in the purified Prb1 fraction was analyzed by MS. (c) Antibody 3008-72 can specifically recognize the truncated H3 starting at Ser22 on Western Blot. Log phase cells of YXY035 (H3 WT, C-HA) and YXY045 (H3 1-21Δ, C-HA) were used as negative and positive control. The whole cell extracts from WT and H3 1-21Δ were separated using SDS-PAGE and analyzed by immunoblotting with antibody 12CA5 (anti HA) and antibody 3008-72 (anti truncated H3). (d) Purified Prb1 and nuclear fraction cleavage site in H3. The H3 endopeptidase activity of purified Prb1 and nuclear extracts from WT cells were assayed with recombinant S. cerevisiae histone H3 and clipping was assayed by western blot with antibody against the truncated H3 (3008-72) and H3 C-terminus (Ab1791).
(PDF)

Figure S2 **Test of H3 endopeptidase activity of GDHs and Prc1.** (a) GDHs are not required for the H3 endopeptidase activity in whole cell extracts of early stationary phase cells. Whole cell extracts from WT, *gdh1*Δ, *gdh2*Δ and *gdh3*Δ were assayed for endopeptidase activity. Clipping was assayed by western blot with antibody against the H3 C-terminus. (b) Prc1 can’t cleave H3 at its N-terminus. Recombinant *X. laevis* histone H3 was incubated with different amount of purified Prc1. Clipping was assayed by western blot with antibody against the H3 C-terminus.
(PDF)

Table S1 **List of yeast strains and plasmids.**
(XLSX)

Table S2 **List of proteins identified by Mass Spectrometry in the HIC fractions.**
(XLSX)
