# Supplementary figures and images for: PRB1 Is Required for Clipping of the Histone H3 N Terminal Tail in Saccharomyces cerevisiae
Source: PLoS One. 2014 Feb 28;9(2):e90496. doi: 10.1371/journal.pone.0090496 (PMC3938757; doi:10.1371/journal.pone.0090496)

Figure S1

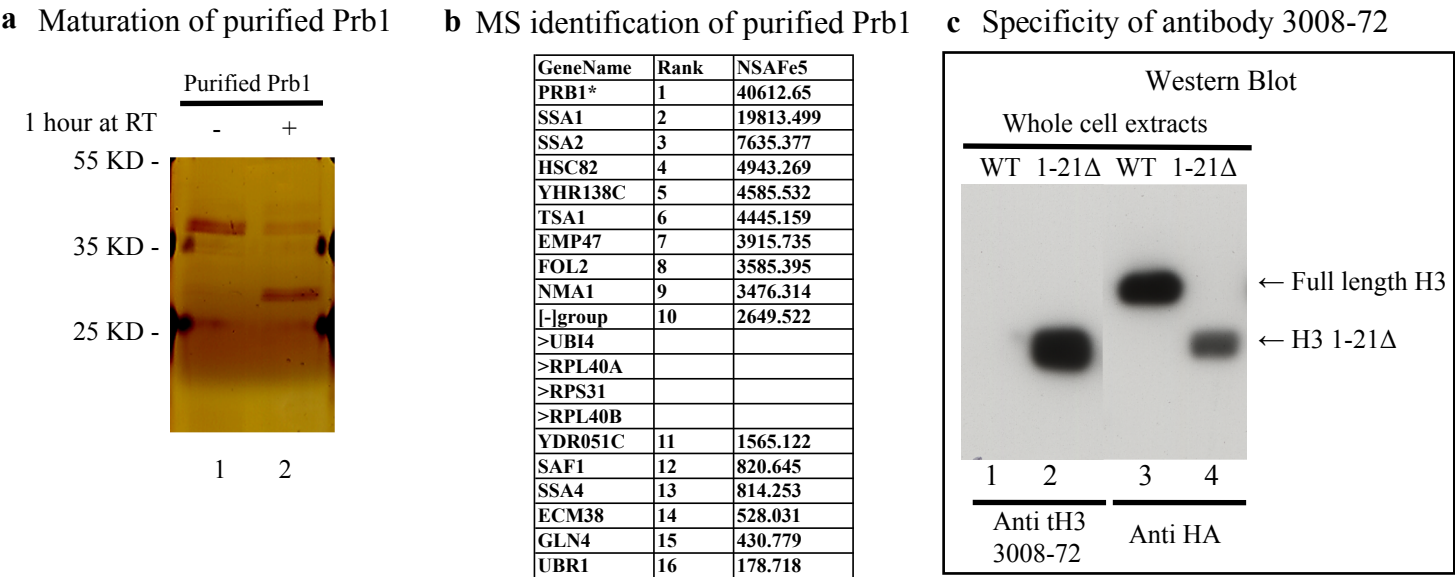

**d** Cleavage site by nuclear fraction and purified Prb1

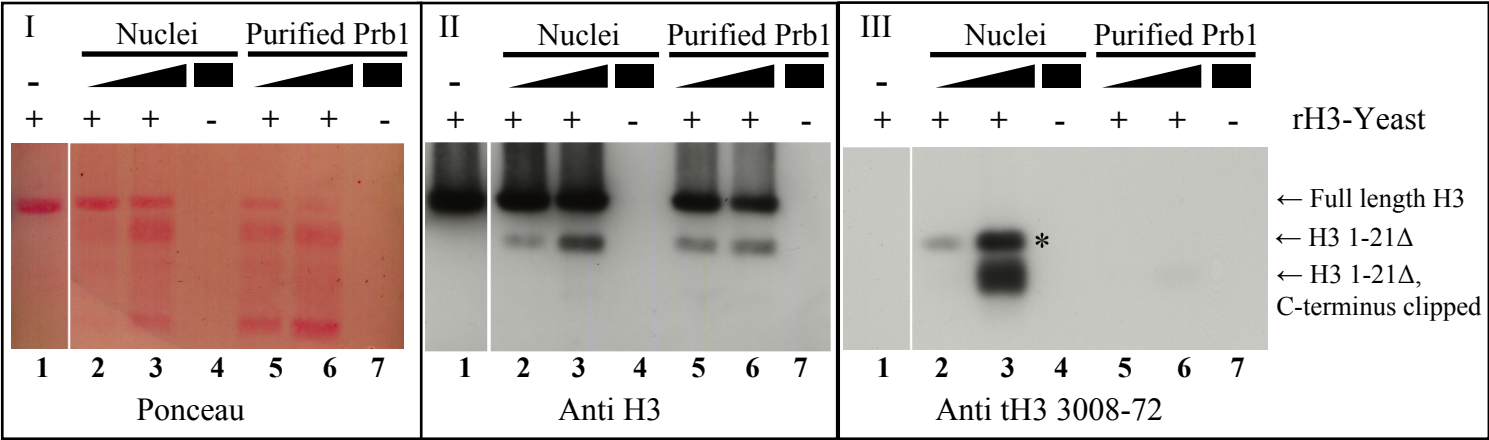

Supplement: Figure S1 — Validation of the purified Prb1, antibody t3008-72 and comparison of the cleavage site of H3 by purified Prb1 and nuclei extract. (a) Proteolysis of purified Prb1. Purified Prb1 was incubated either on ice or room temperature for 1 hour, then separated by SDS-PAGE and stained by silver. (b) Mass spectrometry analysis of the purified Prb1. Prb1 was purified from the strain YSC3869-9518684 (Open Biosystems) containing the PRB1 expression plasmid as described in the Materials and Methods. The abundance of proteins in the purified Prb1 fraction was analyzed by MS. (c) Antibody 3008-72 can specifically recognize the truncated H3 starting at Ser22 on Western Blot. Log phase cells of YXY035 (H3 WT, C-HA) and YXY045 (H3 1-21Δ, C-HA) were used as negative and positive control. The whole cell extracts from WT and H3 1-21Δ were separated using SDS-PAGE and analyzed by immunoblotting with antibody 12CA5 (anti HA) and antibody 3008-72 (anti truncated H3). (d) Purified Prb1 and nuclear fraction cleavage site in H3. The H3 endopeptidase activity of purified Prb1 and nuclear extracts from WT cells were assayed with recombinant S. cerevisiae histone H3 and clipping was assayed by western blot with antibody against the truncated H3 (3008-72) and H3 C-terminus (Ab1791). (PDF) [file pone.0090496.s001.pdf]

**Figure S2**

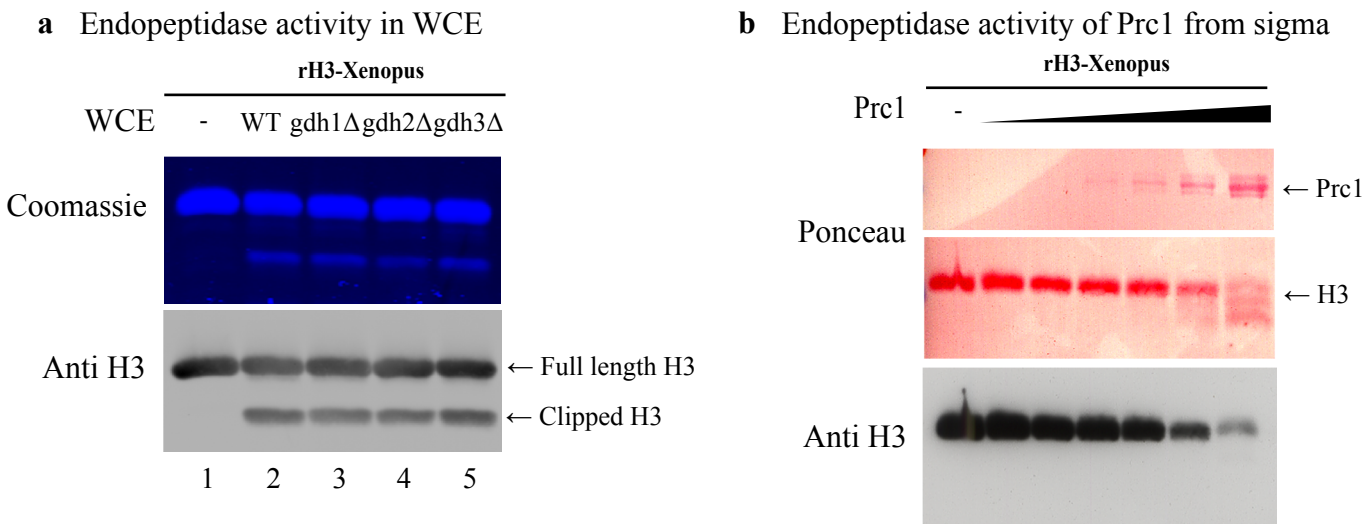

Supplement: Figure S2 — Test of H3 endopeptidase activity of GDHs and Prc1. (a) GDHs are not required for the H3 endopeptidase activity in whole cell extracts of early stationary phase cells. Whole cell extracts from WT, gdh1Δ, gdh2Δ and gdh3Δ were assayed for endopeptidase activity. Clipping was assayed by western blot with antibody against the H3 C-terminus. (b) Prc1 can’t cleave H3 at its N-terminus. Recombinant X. laevis histone H3 was incubated with different amount of purified Prc1. Clipping was assayed by western blot with antibody against the H3 C-terminus. (PDF) [file pone.0090496.s002.pdf]
